# Research on fracture mechanism of rock mass with orthogonal cracks under uniaxial compression

**DOI:** 10.1371/journal.pone.0351735

**Published:** 2026-07-07

**Authors:** Xuehua Li

**Affiliations:** School of Civil Engineering and Architecture, Nanyang Normal University, Nanyang, Henan, China; Henan Polytechnic University, CHINA

## Abstract

The initiation, expansion and penetration of cracks in rock masses are important reasons for rock mass failure. Orthogonal crack is the most typical form of cracks. There is still a lack of in-depth and detailed research on the law of expansion and penetration of orthogonal cracks. In this paper, the RFPA software is used to study the failure process of the rock mass with orthogonal fractures, and the influence of the change of the angle *α* between the main fracture and the loading direction on the failure mode and the failure mechanical properties of the specimen is comprehensively explored. The simulation results show that the angle α between the different main fractures and the loading direction affects the internal stress state of the rock, redistributes the stress field at the tip of the fracture and the surrounding area, resulting in the difference in the energy required for the expansion of the new fracture, which is manifested in the macroscopically as the difference in the strength of the rock and acoustic emission quantity.

## 1. Introduction

The rock mass usually contains three sets of structural planes, namely bedding, face cleats and end cleats, all of which occurred during the diagenesis period. The three sets of structural planes are often distributed vertically, so the distribution of rock mass structural planes has orthogonal characteristics [1–6]. In this paper, the rock mass containing the network of orthogonal structural planes is called the rock mass containing orthogonal fractures.

The initiation, expansion, and penetration of internal cracks in rock masses are one of the main reasons for instability and failure of rock mass engineering. Underground projects that have been built or under construction have encountered a large number of rock masses containing orthogonal cracks [7–9]. In the process of engineering excavation and operation, the disasters caused by the fracture and instability of fractured rock mass have caused serious casualties and economic losses. Therefore, it is of great theoretical significance and engineering value to carry out the research on the fracture mechanism of rock mass with orthogonal fractures, obtain the rock strength and crack evolution characteristics under the state of orthogonal fractures, and realize the stability prediction of fractured rock mass for the selection of deformation control theory and support mode of underground engineering structures [10–12].

Some scholars had studied the failure law of fractured rock mass by means of indoor test. Marek [13] studied a brittle rock specimen containing a single inclined crack. Through uniaxial compression tests, it was found that the crack propagated in the direction of about 70° along the original crack surface. Wong and Einstein [14] studied the crack propagation mechanism of a single-crack marble specimen and observed three types of cracks, including tensile cracks, shear cracks, and tensile-shear composite cracks. Zhao et al. [15] studied the crack propagation process and stress distribution of parallel double cracks under uniaxial compression, and observed that different types of tensile and shear cracks appeared in the crack propagation. Wong and Chau [16] studied rocks with different dip angles of parallel fractures and rock bridge, and obtained the variation law of compressive strength of rock mass with different dip angles. However, the relevant research on fracture mechanism of rock mass with orthogonal fractures under uniaxial compression load is very rare at home and abroad. Yang [9] studied the propagation process of red sandstone fracture specimens under uniaxial compression and the influence of the inclination of the fracture on the strength and deformation parameters. Zhang et al. [17] studied the influence of the angle change between the primary and secondary cracks on the failure mode and mechanical properties of joint specimens with cross cracks. Due to the difficulty of sampling fractured rock masses, physical simulation, theoretical analysis and numerical simulation methods are often used to study the mechanical properties of fractured rock masses [18].

Numerical simulation research can avoid the problem of sample discreteness in physical tests and obtain more systematic crack propagation and penetration laws. It has important reference value for interpreting physical tests of rock mass mechanics and solving engineering problems. In this paper, RFPA software is used to analyze the fracture mechanism of rock mass with orthogonal fractures under uniaxial compression load, and to study the influence of orthogonal cracks on the strength characteristics, deformation characteristics, crack propagation process, and ultimate failure mode of the rock mass.

## 2. Introduction to RFPA software

### 2.1. The basic principle of RFPA

In 1995, Professor Tang proposed the real failure process analysis method, namely the RFPA (Realistic Failure Process Analysis) method. This method is based on the basic theory of finite element and fully considers the characteristics of nonlinearity, inhomogeneity and anisotropy that accompany the rock fracture process.

RFPA is a real fracture process analysis system that uses elastic mechanics as a stress analysis tool and elastic damage theory as a medium deformation analysis module. The basic idea is [19–21]:

(1) The material model is discretized into a numerical model composed of meso-level primitives, and the material medium is an isotropic elastic-brittle or brittle-plastic in the microscopic view;
(2) It is assumed that the mechanical properties of the discretized meso-element obey the Weibull distribution, thereby establishing the relationship between the meso- and macro-medium mechanical properties;
(3) According to the basic element linear elastic stress and strain solution method in elastic mechanics, analyze the stress and strain state of the numerical model.
(4) Introduce appropriate element failure criterion and damage law, and the critical point of elementary transformation obeys the modified Coulomb criterion;
(5) The crack propagation of materials is a quasi-static process, and the influence of inertial force caused by rapid propagation is ignored.

### 2.2. Realization of rock mass inhomogeneity

Due to the inhomogeneity and the randomness of defect distribution in rock mass, complex reflection, diffraction and interference phenomena will occur in the propagation of stress waves in rock media. Therefore, the inhomogeneity of rock mass and its variation law are important factors that must be considered in the study of rock fracture process. RFPA software uses Weibull distribution in statistical mathematics to describe the inhomogeneity of rock mass, as shown in Formula 1.


$$\begin{array}{c} \varphi \left(E\right)=\overset{e}{\underset{\text{0}}{\int }}\phi \left(x\right)dx=\overset{e}{\underset{\text{0}}{\int }}\left(\frac{m}{\alpha_{\text{0}}}\cdot {\left(\frac{\alpha }{\alpha_{\text{0}}}\right)}^{m-1}\cdot e^{-\left(\frac{\alpha }{\alpha_{\text{0}}}\right)}m\right)dx=1-e^{-{\left(\frac{E}{E_{\text{0}}}\right)}^{m}} \end{array}$$ (1)

Where φ(E) is the statistical number of elements with elastic modulus *E*.

A sample space is composed of units formed by the statistical distribution of formula (1). The integral space distribution is different due to the difference of *m* value under the condition of constant mean value *E*_0_. The unit average properties of the material medium composed of these primitives may be roughly the same. However, the elements spatial arrangement is significantly different due to the disorder of microscopic structure. This disorder in the microscopic level just reflects the rock unique discrete characteristics

## 3. Numerical model

**Fig 1:**
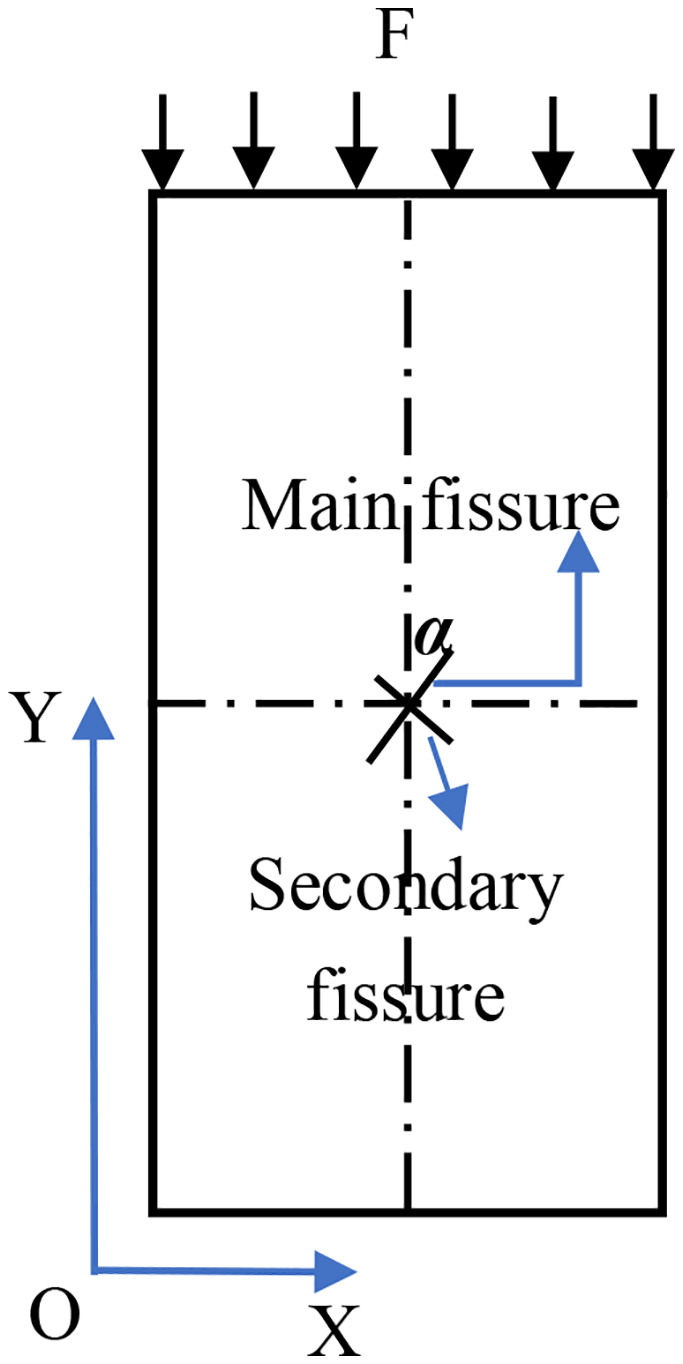
Numerical model.

The numerical simulation software RFPA is used to study the rock mass with orthogonal fractures. The physical and mechanical parameters of the rock mass are shown in Table 1. The mean degree coefficient *m* is the uniformity coefficient of the rock medium. The smaller the value of *m*, the more discrete the mechanical properties of the rock mass. The numerical model of rock mass with orthogonal cracks is shown in Fig 1, and the specimen size is 50 mm × 100 mm, the unit is divided into 100 × 200 = 20000. The fracture with a length of 20 mm is defined as the main fracture, and the fracture with a length of 15 mm is defined as the secondary fracture, the fracture thickness is one unit, and the angle between the main fracture and the secondary fracture is 90°, the orthogonal fracture is located in the center of the numerical model. In Fig 1, *α* is the angle between the main crack and the loading direction, and *α* is taken as 0°, 15°, 30°, 45°, 60°, 75°, and 90°, respectively. This simulation adopts displacement loading method, and the loading rate is 0.002 mm/step. The empty element is used to replace the crack in the numerical simulation. The cohesion, friction angle and tensile strength of the cracks are 0.

**Table 1:** Physical and mechanical parameters of rock mass.

Parameter	Rock
Homogeneity index (m)	2
Mean uniaxial compressive strength (MPa)	200
Mean Young’s modulus (GPa)	60
Poisson’s ratio	0.25
Density (kg/m^3^)	2500
Friction angle (°)	30

## 4. Simulation results and analysis of uniaxial compression

### 4.1. Failure mode analysis

**Fig 2:**
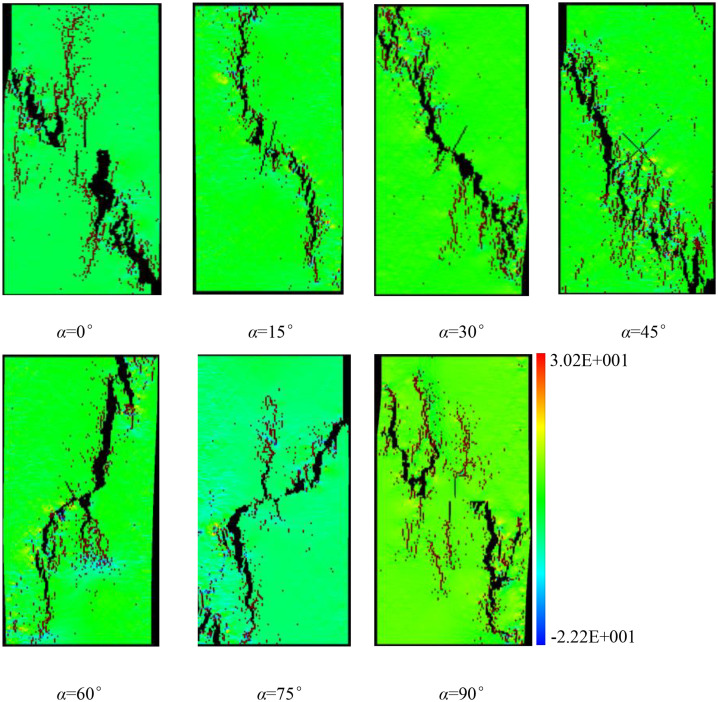
Failure modes of rock masses with different *α* angles.

It can be seen from Fig 2 that there are four failure modes of rock mass with orthogonal fractures when the main fracture and loading direction are different. The first type, when *α* = 0°, 15°, 30°, the rock mass produces a macroscopic fracture surface that penetrates the rock mass along the secondary fissure. The second type, when *α* = 45°, the rock mass produces a macroscopic fracture surface that penetrates the rock mass, and this fracture surface does not occur along the orthogonal fracture. The third type, when *α* = 60° and 75°, the rock mass will produce a through-rock mass fracture surface along the secondary fissure, and at the same time, one end of the main fissure will also be damaged. In the fourth type, when *α* = 90°, the rock mass produces a macroscopic fracture surface that penetrates the rock mass along the secondary fissures, and at the same time, both ends of the main fissures are also damaged to a certain extent.

According to the above analysis, it can be seen that no matter which type of rock mass failure, the rock mass will produce a macroscopic fracture surface through the rock mass under the condition of uniaxial compression loading. This phenomenon indicates that the change of the angle *α* between the main fracture and the loading direction has little effect on the failure mode of the rock mass. This conclusion has a certain guiding significance for engineering practice. There are always a large number of fracture networks in engineering rock mass. For simple stress conditions, no matter how complex the fracture form is, the instability of rock mass is always associated with the expansion and instability of primary fractures.

The fracture process of the four failure modes of the rock mass with orthogonal fractures is described in detail below.

**Fig 3:**
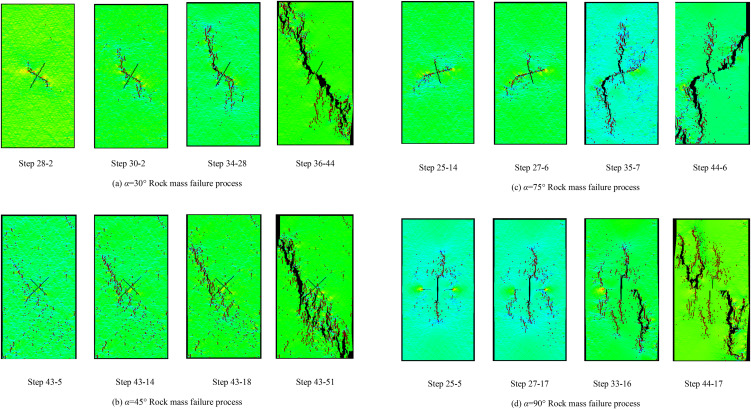
Typical failure process of rock masses with different *α* angles.

The first type is illustrated with *α* = 30° as an example (see Fig 3a). The newly formed fractures are first generated at the tip of the secondary fractures and appear basically symmetrically. Generally, the newly formed fractures are relatively small. With the increase of the load, the new fractures continue to develop toward the end of the rock, and at the same time the new fractures continue to widen, and there are basically no new fractures in other places in the rock.

The second type is explained by taking *α* = 45° as an example (see Fig 3b). First, a tensile crack is induced in a certain tensile stress concentration area inside the specimen. As the external force increases, the tensile crack eventually develops into a macroscopic fracture surface through the rock mass. According to the maximum circumferential tensile stress criterion in fracture mechanics, when *α* = 45°, the maximum shear stress plane usually forms an angle of about 45 ° with the direction of the maximum principal stress. If the direction of the primary crack is not consistent with this dominant failure surface, the macroscopic fracture surface will tend to expand along the direction of maximum shear stress or tensile stress concentration, rather than along the existing orthogonal cracks.

The third type is explained by taking *α* = 75° as an example (see Fig 3c). First, tension cracks are initiated from the tensile stress concentrated areas near the two tips of the secondary cracks. At the same time, the crack also occurs at one end of the main crack, and the direction of the new cracks at one end of the main crack is parallel to the loading direction, but the macroscopic fracture surface that penetrates the rock mass passes through the secondary fissures. The fourth type takes *α* = 90° as an example (see Fig 3d). The fourth fractur mode is basically the same as the third fractur mode, the only difference is that both ends of the main fracture appear cracking.

### 4.2. Stress analysis

**Fig 4:**
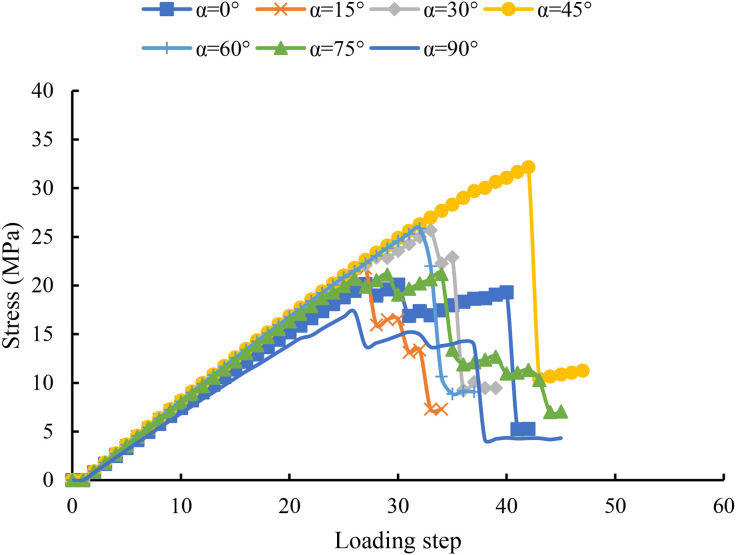
Stress variation curves of rock masses with different *α* angles.

It can be seen from Fig 4 that during the initial loading, the stress-loading step curves of different α-angle rock masses all overlap, and the stress curves all show consistent regularity. With the increase of external force, the stress curve shows a downward trend after reaching the maximum value. Among them, the downward trend of the stress curve with *α* = 45° is the most obvious. At this time, the stress curve shows obvious elasticity and brittleness. When *α* = 75°, the stress curve decreases slowly, at this time, the stress curve shows obvious post-peak softening characteristics.

**Fig 5:**
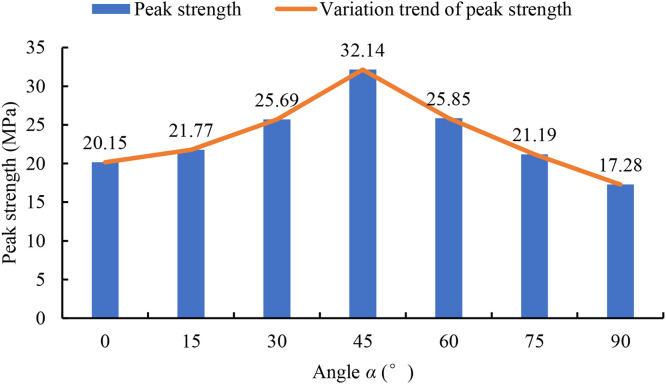
Variation trend of peak strength of rock masses with different *α* angles.

**Fig 6:**
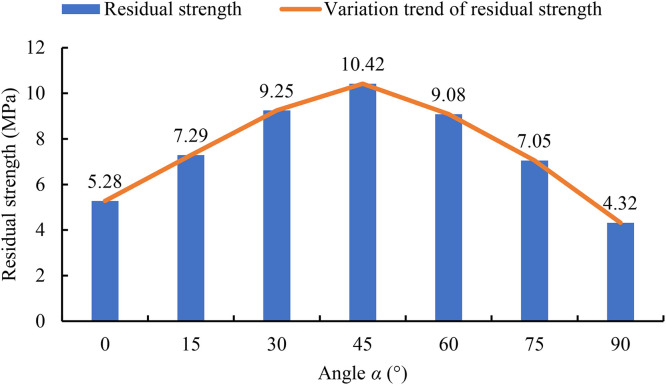
Variation trend of residual strength of rock masses with different *α* angles.

**Fig 7:**
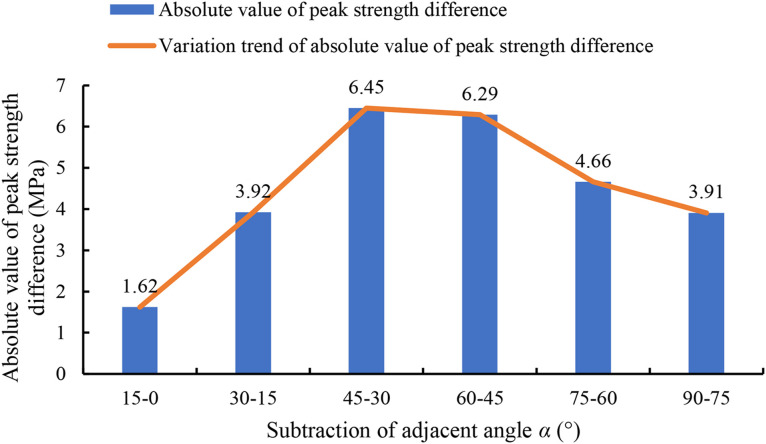
Variation trend of the absolute value of the peak strength difference of adjacent *α* angle rock masses.

**Fig 8:**
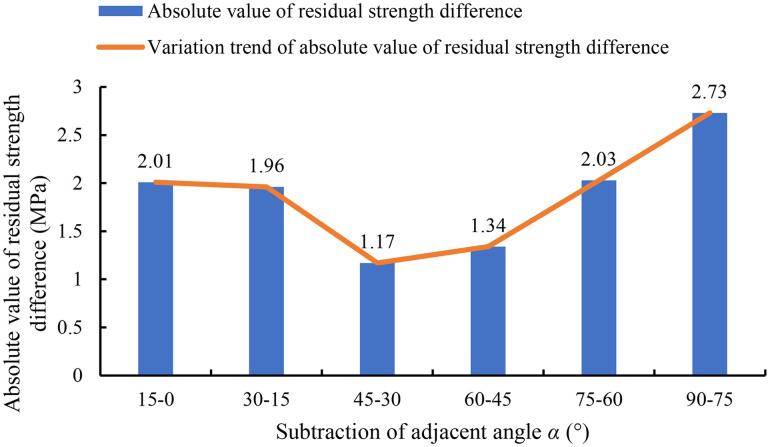
Variation trend of absolute value of residual strength difference of adjacent *α* angle rock mass.

It can be seen from Fig 5 and Fig 6 that when the angle *α* is from 0° to 45°, the peak intensity and residual intensity gradually increase. When the angle *α* is from 45° to 90°, the peak intensity and residual intensity gradually decrease. Moreover, the slope of the peak strength and residual strength curves in the rising stage is almost the same as that in the declining stage. When *α* = 45°, the peak strength and residual strength reach the maximum, which are 32.14 MPa and 10.42 MPa, respectively. When *α* = 90°, the peak strength and residual strength reach the minimum, which are 17.28 MPa and 4.32 MPa, respectively. It can be seen from Fig 7 that the absolute value variation trend of the peak strength difference of adjacent α-angle rock bodies presents a law of first rising and then falling. It can be seen from Fig 8 that the absolute value variation trend of the residual strength difference of adjacent α-angle rock masses decreases first and then increases.

According to the above analysis, it can be known that the different angle *α* between the main cracks and the loading direction affects the stress field of rock mass after being loading, thereby changing the bearing capacity of the samples and causing the difference of compressive strength of rock mass.

When the crack surface slides, the local stress field near the crack tip will redistribute. Under the condition of *α* = 45°, due to the maximum shear stress and sliding displacement, a high amplitude tensile stress zone (perpendicular to the direction of the maximum principal stress) is generated in front of the crack tip. This stress concentration can trigger the initiation and propagation of secondary cracks. But during the propagation process, the direction of the principal stress gradually deviates, causing the crack to tend to turn parallel to the loading direction (known as “wing shaped cracks”). Throughout the process, the stress redistribution range is large and the energy dissipation is high, thereby enhancing the macroscopic peak strength [22–25].

In contrast, when *α* = 90°, the crack surface is closed under pure compression, and there is almost no shear stress driving sliding near the tip. The stress redistribution is limited to the elastic compression zone. Once the transverse tensile stress exceeds the material’s tensile strength, unstable splitting occurs instantly, resulting in low strength brittle fracture.

### 4.3. Acoustic emission analysis

**Fig 9:**
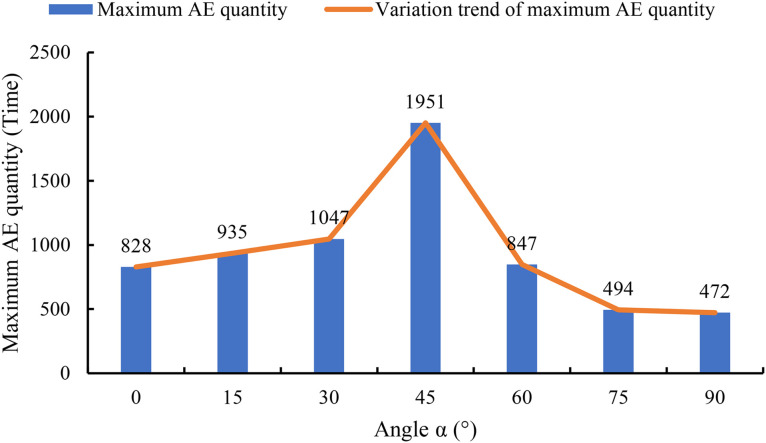
Variation trend of the maximum AE quantity of rock masses with different *α* angles.

**Fig 10:**
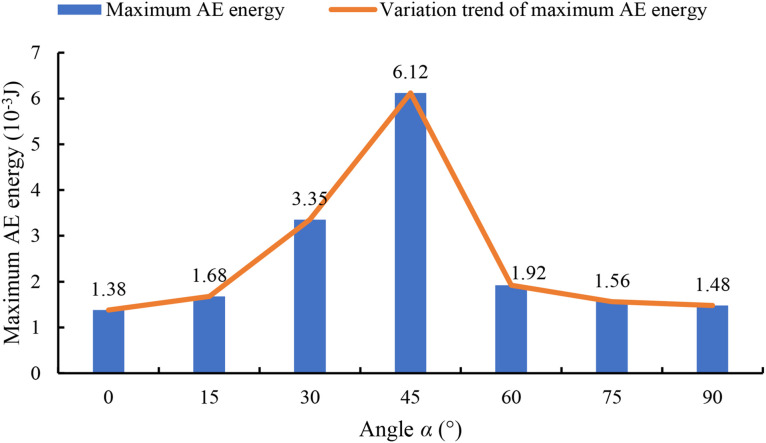
Variation trend of the maximum AE energy of rock masses with different *α* angles.

Acoustic emission (AE) is the phenomenon of rapid release of strain energy in the form of elastic waves during the initiation, propagation, and fracture of internal microcracks in materials. After the numerical simulation is over, the RFPA software can automatically display the AE energy, the cumulative total energy, AE quantity and the cumulative total quantity during the loading process. From Fig 9 and Fig 10, it can be seen that when the angle *α* is from 0° to 30°, the maximum AE quantity slowly increases. When the angle *α* is from 30° to 45°, the maximum AE quantity increases sharply. The largest AE quantity is 1951. When the angle *α* is from 45° to 60°, the maximum AE quantity decreases sharply. When the angle *α* is from 60° to 90°, the maximum AE quantity slowly decreases, and the maximum AE quantity reaches the lowest value of 472. The maximum AE energy is basically the same as the maximum AE quantity. When the angle *α* is 45°, the maximum AE energy reaches the maximum value of 6.12 × 10^−3^ J. When the angle *α* is 0°, the maximum AE energy reaches the minimum value of 1.38 × 10^−3^ J.

**Fig 11:**
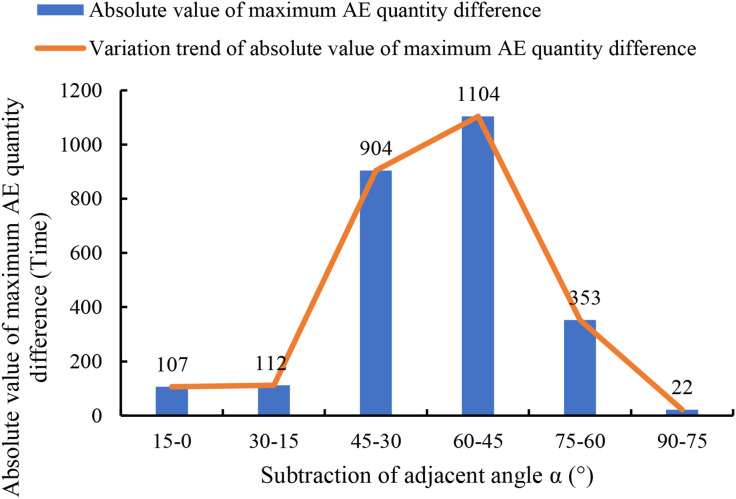
Variation trend of the absolute value of the maximum AE quantity difference of adjacent *α*-angle rock masses.

**Fig 12:**
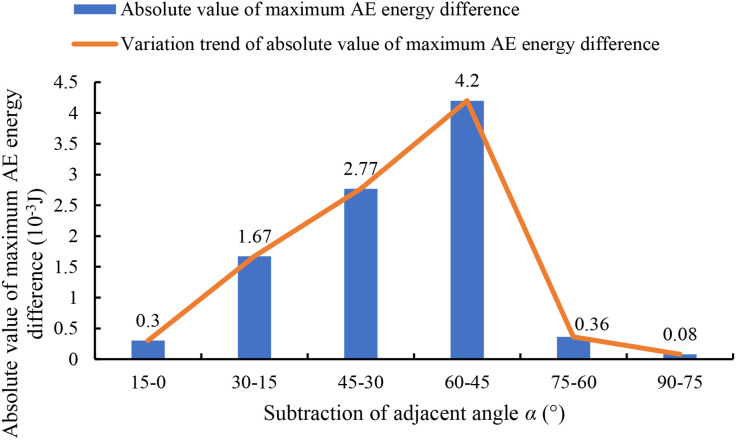
The absolute value change trend of the maximum AE energy difference of adjacent *α*-angle rock masses.

From Fig 11 and Fig 12, it can be seen that the absolute value of the maximum AE quantity difference and the absolute value of the maximum AE energy difference of adjacent *α*-angle rock masses both show the law of first rising and then falling. Moreover, the rising and falling slopes of the two curves are relatively large.

**Fig 13:**
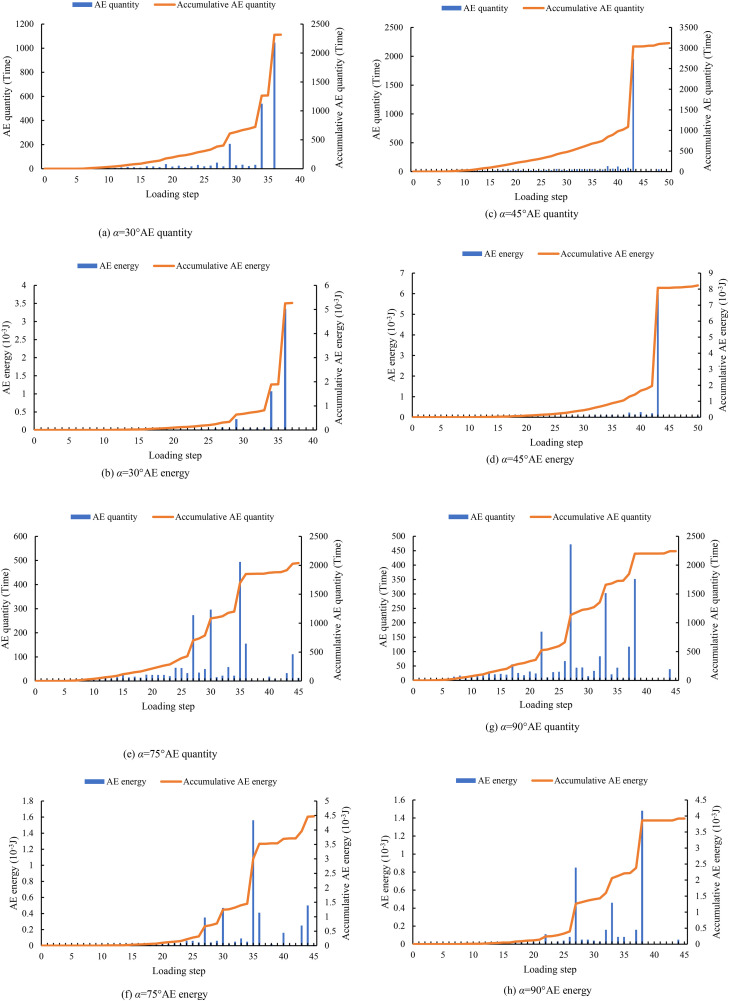
The relationship between the typical AE quantity and AE energy of rock masses with different *α* angles and the loading step.

Fig 13 shows the variation law of AE quantity and AE energy corresponding to four typical failure modes. In the first failure mode (taking *α* = 30° as an example), there will be two increasing peaks before the AE quantity reaches the maximum value, and the AE cumulative curve shows two obvious sudden increases. AE energy and AE quantity show the same law. The second damage mode (taking *α* = 45° as an example), the AE quantity increases and decreases abruptly, there is only one peak in the AE quantity, and the AE cumulative curve shows obvious jump phenomenon, the AE energy and AE quantity show the same law. The third failure mode (taking *α* = 75° as an example) and the fourth failure mode (taking *α* = 90° as an example) have similar variation law in AE quantity. there are obvious peaks before and after the maximum value of AE quantity and AE energy, and the distribution of AE quantity and AE energy are relatively discrete.

From the above analysis, it can be seen that for the samples of the same size and form, different fracture distribution forms are the main reasons for the difference of energy required for fracture initiation and rock mass instability. On the macro level, it is shown that the characteristics of AE quantity and AE energy are different.

## 5. Conclusion

(1) The failure mode of the rock mass is tensile failure, the fracture surface of the rock mass presents a certain angle with the loading direction. The crack initiation position of the fracture surface occurs at the tip of the secondary fracture, and the secondary fracture occurs at the tip of the main fracture.
(2) The stress-loading step relationship curves of rock masses with different *α* angles generally show elastic brittleness or elasticity-post-peak softening characteristics. When the angle *α* between the main crack and the loading direction changes, the stress-loading step-post relationship shows different laws.
(3) The peak strength of rock mass, residual strength, maximum AE quantity, and maximum AE energy all reach the maximum when *α* = 45°.The absolute value of the peak intensity difference of adjacent *α*-angle rock masses, the absolute value of the maximum AE quantity difference, and the absolute value of the maximum AE energy difference also show a trend of first increasing and then decreasing. The absolute value of the residual strength difference of adjacent *α*-angle rock mass shows a trend of first decreasing and then increasing.
(4) Under the critical condition of *α* = 45°, the actual fracture surface often deviates from the joint surface and turns towards a path that intersects with the direction of the maximum principal stress at a small angle. Therefore, the engineering reinforcement design (such as anchor direction, support strength) and stability calculation should fully consider this deviation effect.

## Supporting information

S1 File Data. (XLS)
